# The Effect of Memory in Inducing Pleasant Emotions with Musical and Pictorial Stimuli

**DOI:** 10.1038/s41598-018-35899-y

**Published:** 2018-12-05

**Authors:** Johanna Maksimainen, Jan Wikgren, Tuomas Eerola, Suvi Saarikallio

**Affiliations:** 1 Department of Music, Art and Culture Studies, University of Jyvaskyla, Jyvaskyla, Finland; 2 Department of Psychology, University of Jyvaskyla, Jyvaskyla, Finland; 3 Department of Music, Durham University, Durham, United Kingdom

## Abstract

Music is known to evoke emotions through a range of mechanisms, but empirical investigation into the mechanisms underlying different emotions is sparse. This study investigated how affective experiences to music and pictures vary when induced by personal memories or mere stimulus features. Prior to the experiment, participants were asked to select eight types of stimuli according to distinct criteria concerning the emotion induction mechanism and valence. In the experiment, participants (*N* = 30) evaluated their affective experiences with the self-chosen material. EEG was recorded throughout the session. The results showed certain interaction effects of mechanism (memory vs. stimulus features), emotional valence of the stimulus (pleasant vs. unpleasant), and stimulus modality (music vs. pictures). While effects were mainly similar in music and pictures, the findings suggest that when personal memories are involved, stronger positive emotions were experienced in the context of music, even when the music was experienced as unpleasant. Memory generally enhanced social emotions specifically in pleasant conditions. As for sadness and melancholia, stimulus features did not evoke negative experiences; however, these emotions increased strongly with the involvement of memory, particularly in the condition of unpleasant music. Analysis of EEG-data corroborated the findings by relating frontomedial theta activity to memory-evoking material.

## Introduction

The induction of affect is a multidisciplinary topic that intrigues scholars from psychology, cognitive neuroscience, and art. Due to its complexity, affect induction requires approaches ranging from laboratory experiments to subjective evaluations of personal meanings, to generate comprehensive knowledge of affect induction principles. In the studies of emotions induced by art objects, however, the participants’ personal memories, experiences, and meanings constitute an area that often remains beyond reach in laboratory settings. Furthermore, while some modality specific features have been identified in the perception of auditory and visual stimuli^1^, little is known about cross-modal differences of affective processing involving personal meanings and memories. This study aims to find out (a) whether emotions involving personal memories impact the affective experience and (b) whether the potential influence of memory is similar in distinct modalities (music and pictures) and in pleasant and unpleasant experiences.

## Pleasant and Unpleasant Emotions

In the studies scrutinizing positive and negative emotions, valence refers to the intrinsic attractiveness (positive valence) or aversiveness (negative valence) evoked by an object. *Affect*, considered as an umbrella term, covers all evaluative (positive/negative) states, such as mood, and aesthetic evaluation (see^2^) – or to put it more generally, all the positive and negative evaluative states^3^. The term *valence* is used here to describe specific emotions on a dimension ranging from *pleasurable to unpleasant*. More broadly, valence is part of a two-dimensional model of emotions^4^ that proposes that all affective states arise from independent neurophysiological systems, one related to valence and another related to arousal. The model is widely adopted in affective sciences^5,6^, albeit not the only candidate for the emotions^7^.

There are several competing hypotheses as to why distinct modalities, such as music and pictures, may evoke strong positively valenced experiences. For instance, *peak sensations* that are typically studied through self-reports, physiological responses, and by neural measurements, have been studied in the context of music and have been associated with the release of dopamine via the neural reward system^8–10^. Related sensations are often depicted as chills, shivers, or goose bumps, and when music is involved, they seem to correspond, for instance, to unexpected changes or gradual expansions in the dynamics, structure, and volume of music (see, for example^11–16^). Assuming a close link between pleasant feelings and affective arousal in the past studies, one assumes that self-reports and EEG-measures reliably evince the respondents’ affective engagement with music and pictures.

The experiences related to pleasure as characterized by extreme positive valence could be characterized in several ways; top-down influences such as activation of specific memories and symbolic construction are assumed to be important generators of pleasure^17–20^. In such instances, emotional experience cannot be explained by reducing them to stimulus properties, such as musical structure or visual elements^21,22^. The simultaneous presence of distinct emotions may further result in *mixed affective* experiences. Exposure to negative emotions occurring in art reception is often associated with positive emotions, constituting a so-called *paradox of art*^23^. For example, sadness, that is typically experienced in the context of music^24,25^ – defined as a negative emotional state in psychology^25 (see also26^) – may be experienced as pleasant when associations and memories are strongly involved^27^ (further details about enjoyment of negative emotions across different fields of art, see e.g.^28^). Instead, when the stimulus is conceived as purely negative it activates defensive motivation systems found to enhance sensory, perceptual, and memory processes^29,30^, leading to displeasure, also frequently elicited by unfamiliar or violent music^31^ (for visual arts, see^32^).

## Emotion Induction Mechanisms

Emotion induction mechanisms cover a range of processes, such as perception, memory, imagination, and somatic experience, as well as those behavioral processes that result from the elicitation of emotion. The term *induction mechanism* is used in the present study to broadly refer to all information processing that leads to the induction of emotions through the perception of the object^3^.

Previous studies have identified several distinct induction mechanisms, some focusing on fewer possibilities, such as *cognitive appraisal*^33^, and others presenting an extensive set of mechanisms and emotion induction moderators^14,34,35^. The empirical investigation of these mechanisms is sparse; some mechanisms, such as *expectancy*, appear to be invoked less often than other mechanisms, such as *episodic memory*^2^. This may relate to the fact that implicit mechanisms remain unacknowledged in the studies based on self-reports. Also, some mechanisms may be more suitable for inducing particular emotions; music-induced sadness, for instance, may use the feature-based mechanism of *emotional contagion* while happiness may be induced by memories^36^. Another assumed asymmetry between emotions and induction mechanisms relates to emotions that are strongly social such as tenderness. Social emotions are likely to relate to social connectedness and belonging^37–40^. It is expected that such emotions are linked with different induction mechanisms, although no such studies have yet been carried out.

## Comparison of Distinct Modalities

Most studies of pleasurable music have focused on sedentary listening. In that respect these findings may relate to pictures as well, and indeed, emotion induction theory^14^ assumes that music evokes emotions through mechanisms that are not unique to music. There is, however, relatively little prior research directly comparing music and pictures as modalities for emotional experiences. While music has been actively studied as a source for distinct emotions and daily pleasure, research on pictures has rather focused on aesthetic appreciation of visual stimuli as cognitive-perceptual judgments^41^.

Previous research suggests some modality-based qualitative differences in the elicited emotional experiences. Rottenberg and her colleagues^42^ proposed the following key dimensions that differentiate emotion induction: (1) emotional intensity, (2) complexity, (3) levels of attentional capture, (4) demand characteristics, (5) standardization, (6) temporal considerations, and (7) ecological validity. The dimensions may vary in terms of their relevance depending upon the variable under scrutiny. For example, music may score high in ecological validity through the activation of associated memories, the elicitation of imagery, and conditioned emotional responses^14^; regarding pictorial materials, the personal relevance and ecological validity dimensions may be lower due to the related approach-avoidance motives to a minor extent, than real-life interactions^43^. This suggests that emotions evoked by visual arts may be perceived as less similar to emotions experienced in other everyday life situations.

The present study focuses on comparable aspects of the phenomenon (emotion, valence, mechanism) that are present and hold relevance for both modalities, yet we assume that there are fundamental variations between modalities due to the differences in the way they relate to space (picture) and time (music), and the generic conventions of practice. Despite the issue of comparability, both music and images are able to evoke a diverse set of emotions and are found to be highly effective for inducing emotional states^43–46^, driven by hedonic expectations and reward^47–50^, or more generally, positive affect^44^. To our knowledge, past research has not attempted to clarify whether emotions induced by images tap into similar mechanisms as emotions induced by music. Furthermore, if the targeted emotion is the same across the domains, such as pleasure or displeasure, do the two domains have distinct ways of inducing these states?

## Rationale

The aim of the study was to explore the interaction effects between stimulus valence, emotion induction mechanism, and modality. The specific focus of interest was to investigate the role of memory as a mechanism for inducing pleasant emotions. This focus can be divided into two research questions related to memory as the emotion induction mechanism:

1. Do episodic memories *contribute to emotional intensity?* Are the induced emotions stronger if they carry personal memories when compared to emotions induced by stimulus features^51,52^?
2. Do episodic memories lead to similar emotional valence in both domains? In other words, *is there a three-way interaction among the emotion induction mechanism, stimulus valence, and stimulus modality*?

### Study design and hypotheses

Differences between pleasant and unpleasant emotional experiences of music and pictures representing four stimuli types that differed in terms of emotion induction mechanism (memory vs. stimulus feature) and valence (pleasant vs. unpleasant), were examined using self-reports and neurophysiological measures. All stimuli were participant-selected (see e.g.^8,10^), and a within-subject design was utilised.

Our hypothesis concerning emotion induction mechanisms is that e*motions involving memories are more intense than experiences based on stimulus features*. Art reception involves interactions of stimulus characteristics, individual differences, and context^53–56^. Research on highly intense emotional responses to art has revealed a close connection between personal memories and emotional responses^10,15,16,27,57–60^. Such responses may indicate high levels of emotional intensity, high levels of aesthetic enjoyment and high memorability as associated with artworks, the processing of which may also include negative affect. It is expected that emotions induced by memory enhance their intensity more than stimulus features^2,22^. There are also implications of a connection between particular emotions, such as sadness, and strong involvement of memories and associations^61^.

The second hypothesis concerns the modality and valence of emotions. We do *not expect a main effect of modality, but we do expect to observe an interaction between valence, mechanism and modality*. Since both visual and auditory stimuli have been found to elicit strong positive and negative emotions^62,63^, priority for either of the modalities is not assumed. Instead, valence-specific biases to modality are expected to occur. Emotions elicited by negatively valenced (unpleasant) stimuli may differ based on distinctions between modalities. This might be expected due to the knowledge of high ecological validity of music through the activation of associated memories^14^, specifically in the context of negatively valenced emotions^63^. In contrast, looking at pictures may activate motives (e.g., an abstract scene, or Christ on a cross) to a minor extent than real everyday transactions, proposing probable lower personal relevance^43^. Thus, while memories have been identified as an important element for music-induced sadness, the same argument cannot be drawn based on the extant literature on pictures, although some past studies have documented the occurrence of both sadness^64,65^ and the involvement of self-referential memorizing^66,67^ in the context of emotions induced by visual art.

## Methods

### Participants

Thirty participants were recruited through University mailing lists. Those with previously diagnosed mental disorders and those currently under medication for any mental health or mood disorders were excluded. The participants were asked to report about such disorders and/or medication in the beginning of recruitment process, and again in the questionnaire that they filled before the laboratory experiment. Regarding the sample, 77% were women and the mean age was 29 years (*SD* = 5.84), ranging from 21 to 45 years. The participants were given two movie tickets for their participation and provided informed consent prior to the study.

### Stimuli

Stimulus selection strategies were designed to optimise ecological validity and experimental control. Participants were instructed to choose eight stimuli before the experiment: Four pictures and four pieces of music. The emotion induction mechanism was manipulated by altering the stimulus selection principle to rely on (a) personal memory or (b) purely stimulus properties. Valence was varied by asking them to choose either (a) pleasurable or (b) unpleasant stimuli. Both requisites were applied to music and pictures. Therefore, the stimulus instructions were as follows:

*Choose a piece of music*….

- *that evokes pleasure which is based on your personal memories*
- *that evokes pleasure which is based purely on how it sounds*
- *that evokes unpleasantness/aversive emotions based on your personal memories*
- *that evokes unpleasantness/aversive emotions based purely on how it sounds*

*Choose a picture*…

- *that evokes pleasure which is based on your personal memories*
- *that evokes pleasure which is based purely on how it looks*
- *that evokes unpleasantness/aversive emotions based on your personal memories*
- *that evokes unpleasantness/aversive emotions based purely on how it looks*

These materials had to represent an object that could be available for anyone, excluding, for instance, personal photos, and non-published musical productions. The difference between perceived and experienced emotions was explained to the participants before the stimulus selection and again at the beginning of the experiment. They were instructed to focus on their experience rather than on what the music or picture is representing. Participants were instructed to give detailed information about the stimuli to ensure that the researchers found the exact same stimuli. Musical excerpts were downloaded by the experimenters and edited into 40-second excerpts. The segments were chosen by a music expert to be a representative and memorable section of the song. Visual materials were downloaded and each picture was presented in the experiment for 40 seconds to eliminate confounds created by varying durations between the two domains.

#### Stimulus self-reports for validating stimulus selection concerning valence

In the experiment, participants rated verbally the valence of each stimuli during the breaks between stimulus presentation. A scale ranging from −3 (extremely unpleasant) to 3 (extremely pleasant) was used. The ratings were subjected to repeated-measures ANOVA to check assumed pleasantness and unpleasantness across the Valence, Mechanisms, and Modality. Significant effects of Valence (F (1,29) = 435.5, p < 0.001), Modality (F = 9.68, p < 0.05), and Mechanism (F = 9.00, p < 0.05) were observed. Ratings for positively valenced stimuli were higher (M = 1.98, SD = 0.83) than for negatively valenced stimuli (M = −1.47, SD = 1.14). These analyses were essentially a manipulation check. The results corroborate that the stimulus selection principles operated according to intentions.

#### Stimulus properties

The stimulus properties were analysed with computational models. The musical and acoustic qualities of the excerpts were estimated using MIR toolbox^68^ by focusing on a limited set of acoustic features (mean and standard deviation of dynamics, tempo, pulse clarity, register, major-minor, rhythmic fluctuation) that have been shown to be relevant for emotional expression (e.g.^69^). The extraction parameters were like those reported in past studies and the windowed analyses of the features were aggregated using means and standard deviations; these were entered into a two-way repeated-measures ANOVA with Valence and Mechanisms as the factors. Only one feature, spectral flux, showed significant main effects of Valence (F (1,29) = 4.34, p < 0.05) and Mechanism, F (1,29) = 4.47, p < 0.05). The excerpts chosen to represent stimulus feature category (in contrast to memory) in the unpleasant condition were considerably higher in spectral flux; this was also evident in the type of music genres and bands chosen for the unpleasant and unfamiliar condition (typically heavy rock and related genres). The rest of the features did not differ across the selections.

The 120 images were analysed in terms of their overall colour profiles and its complexity. For this, the perceptual hue, saturation, and brightness (HSV) were extracted from the RBG values. The HSV profiles of the images were summarised by the mean and entropy for saturation and brightness histograms, which were binned to 50 values within saturation and brightness distributions. The mean hue values were estimated after conversion into degrees and taking the mean angle of the resulting distribution. The means and entropies of the profiles were subjected to repeated-measures ANOVA across Valence and Mechanism factors, yielding non-significant main effects (F < 2.06 for all six features). In sum, the surface characteristics of the images do not distinguish the sample categories. Next, the images were subjected to automatic content analysis where the tags describing the images were retrieved using an online service (Clarifai). The top 10 tags for each image were then subjected to sentiment analysis using vocabulary approach^70^ based on 13,915 English words rated for Valence, Arousal and Dominance. Each image obtained a score in each dimension based on the average of the matching keywords (8–11 keywords/image). A repeated-measures ANOVA for Valence score revealed significant effects for Valence (F (1,29) = 7.93, p < 0.01) but not for Mechanism (F = 0.46), showing higher Valence scores (M = 6.32, SD = 0.44) for Pleasant images than for Unpleasant images (M = 6.05, SD = 0.54). The Arousal scores exhibited a similar pattern where unpleasant images scored significantly (F = 5.89, p < 0.05) higher Arousal scores (M = 4.22, SD = 0.36) than pleasant images (M = 4.07, SD = 0.34). The dominance scores did not differ across the Valence and Mechanism (F < 1.7). Although the analysis provides an expected summary of the affective themes in the images, it was not sensitive to all culturally appropriate meanings in the images.

### Measures

#### Questionnaire

Self-reports of emotions evoked were collected through a questionnaire prior to the laboratory experiment using 10 emotion concepts for each picture and music excerpt. The selection of concepts was based on factors that resulted from an analysis of both a pilot survey (N = 109) designed to explore the affective characteristics of everyday emotions of music and pictures, and previous studies on emotional responses to music^71,72^. Factor analyses were executed for music and pictures separately, and factors that occurred for both modalities were included in the present study. The factors were labeled as joy, strength, sadness, relaxation, tenderness, eroticism, melancholia, spiritualness, curiosity, and kinship. Participants were instructed to rate the felt intensity of each emotion on a 7-point Likert scale for each of the eight stimuli. High values are indicative of increased intensity of emotional experience (Appendix 1).

#### EEG recordings and analyses

64-channel EEG (BioSemi Active II amplifier system utilizing Active View 6.05 recording software) with a sampling rate of 500 Hz was recorded continuously using active Ag/AGCl electrodes (BioSemi Headcap). As per recommendations of the manufacturer, the electrode voltage offsets were kept below 25 mV. Analyses were executed with Brain Vision Analyzer software (Brain Products) and custom written Matlab scripts. Bad channels were removed and the remaining EEG signals were first re-referenced to the average of all channels and then band-pass filtered from 0.5 to 30 Hz. A segment of 60 s of spontaneous EEG from the early phase of the session was fed into independent components analysis (ICA) to recalculate the data, and minimize the effects of eye blinks and eye movements. ICA (Infomax algorithm) was set to produce as many components as there were channels. Among the first components, there were, in most cases, two components corresponding to eye blinks (clear frontal distribution) and lateral eye movements (sinks and source bilaterally in the anterior part of the head); their contribution to the data were calculated away. The obtained signal was then visually compared with the raw signal to verify that this procedure removed stereotypical artefacts.

The EEG signal was segmented based on the event types. Fast Fourier transformation was applied to an EEG epoch consisting the time window of 2–28 s after the onset of the stimulus. Fourier transformed signals were then averaged across event types. For further analyses, frontomedial (C1, C2, Cz, F1, F2, FC1, FC2, Fz), left frontal (C3, C5, F3, F5, F7, FC3, FC5, FT7) and right frontal (C4, C6, F4, F6, F8, FC4, FC6, FT8) electrode pools were formed by averaging the frequency distributions of these signals. The mean magnitude of theta (4–8 Hz) and alpha (9–13 Hz) frequency band activity was calculated for each participant during each event type. The data of two participants were excluded due to problems related to EEG-recordings.

### Procedure

All experimental protocols were approved by the University of Jyvaskyla Ethics Committee. The methods were carried out in accordance with *the ethical principles of research in the humanities and social and behavioural sciences* defined by the National Advisory Board on Research Ethics in Finland (TENK). Informed consent was obtained from all participants. Participants were given a personal identification code after they provided the stimulus materials; with this code they received access to the questionnaire that was asked to be completed in the two days before neurophysiological measures. Upon arriving for the experiment, participants signed the consent form. They were then seated, facing a monitor. Audio stimuli were presented through stereo speakers and visual stimuli on a PC monitor. All the tasks and instructions were provided through e-Prime 2.0 Professional software operated by the researcher. EEG-data were collected using ActiView lab software. The experiment comprised eight blocks that contained 40-sec presentations of each stimulus type; within each block the order of stimulus presentation was randomized. After each 40-sec excerpt the participants reported orally the level of valence evoked by the stimuli to minimally disrupt ongoing EEG-measurement using scales ranging from −3 (extremely unpleasant) to 3 (extremely pleasant). Each stimulus type was evaluated eight times during the experiment.

### Appendix

Research data is available at Harvard Dataverse open access 10.7910/DVN/ZZR7WX.

## Results

The statistical analysis of the questionnaire data focused on main effects and interactions of the main variables. An overall view on how the particular emotions were rated throughout different conditions was first summarized by mean values. The lowest-scoring emotions (eroticism, spirituality, curiosity) were omitted from further analyses, except sadness, which is in the scope of interest of this study. The emotions *relaxation* (mean 3.29, SD 0.53), *strength* (mean 3.27, SD 0.67), *joy* (mean 3.23, SD 0.64), *tenderness* (mean 3.16, SD 0.65), *kinship* (mean 2.97, SD 0.90), *melancholia* (mean 2.94, SD 0.79), and *sadness* (mean 2.66, SD 0.69), were retained for further analyses. Then, a four-way MANOVA was executed for identifying significant main effects of all variables. A three-way MANOVA was then performed separately for each emotion to explore their main effects and interactions between mechanism, modality, and stimulus valence. EEG-data were analysed to verify the effect of induction mechanism on distinct conditions. Self-evaluations of stimulus valence during the EEG ratings were compared by the original ratings and means per factor between modalities and stimulus types. In addition, both stimulus types were analysed in terms of their acoustic, visual and semantic content that revealed the expected differences across valence (see Methods/Supporting Information).

### Variable main effects and interactions

The repeated four-way MANOVA for *modality, mechanism, valence*, and *emotion* indicated a significant main effect for all variables with several variable interactions (Table 1). All two- and three-way interactions with *emotion* were significant, and thereby constituted grounds for exploring the results separately over singular emotions.

**Table 1:** Summary of ANOVA analyses across the four factors.

	df	F	p	${\eta }_{p}^{2}$
modality	1,29	4.958	0.034	0.146
mechanism	1,29	46.070	0.000	0.614
valence	1,29	763.674	0.000	0.963
emotion	6,174	4.451	0.000	0.133
modality × mechanism	1,29	7.022	0.013	0.195
modality × valence	1,29	0.111	0.742	0.004
mechanism × valence	1,29	0.132	0.719	0.005
modality × mechanism × valence	1,29	4.715	0.038	0.140
modality × emotion	6,174	2.761	0.014	0.087
mechanism × emotion	6,174	5.825	0.000	0.167
modality × mechanism × emotion	6,174	2.326	0.035	0.074
valence × emotion	6,174	68.622	0.000	0.703
modality × valence × emotion	6,174	3.904	0.001	0.119
mechanism × valence × emotion	6,174	7.616	0.000	0.208
modality × mechanism × valence × emotion	6,174	1.839	0.094	0.060

### Main effects and interactions over specific emotions

Three-way MANOVA analyses for modality, mechanism, and valence were performed separately for each emotion. In the following sections emotions are presented in groups labeled as *positive* (joy, strength, relaxation), *social* (kinship, tenderness), and *negative* (sadness, melancholia) emotions. The thematic division is based on theoretical consideration of the functional roles of the emotions, for instance, social emotions relates to social connectedness and belonging^37–40,73^. The division was further supported by observations of similarities in how the interactions were distributed in the data.

#### Positive emotions

For *joy*, significant main effects were observed with *modality* (F (1, 29) = 5.24; p = 0.030), *valence* (F (1, 29) = 306.49; p < 0.001), and *mechanism* (F (1, 29) = 5.63; p = 0.024). Joy was experienced more to *music* than to pictures, more through *memory* than stimulus features, and more to *pleasan*t than unpleasant stimuli. A significant interaction was observed for *modality* × *valence* (F (1, 29) = 4.56; p = 0.041), with the predominance of positively valenced stimuli as a source of joy being even clearer in music than in pictures. Significant interaction was also found for *mechanism* × *valence* (F (1, 29) = 5.66; p = 0.024) with the prevalence of positively valenced stimuli as the source of joy being even clearer when memories were the induction mechanism.

Considering *strength*, significant main effects of *modality* (F (1, 29) = 8.96; p = 0.006), and *valence* (F (1, 29) = 484.58; p < 0.001) were observed. Strength was experienced stronger when evoked by music, and more typically when related to positively valenced stimuli. Significant interactions were not found.

**Figure 1:**
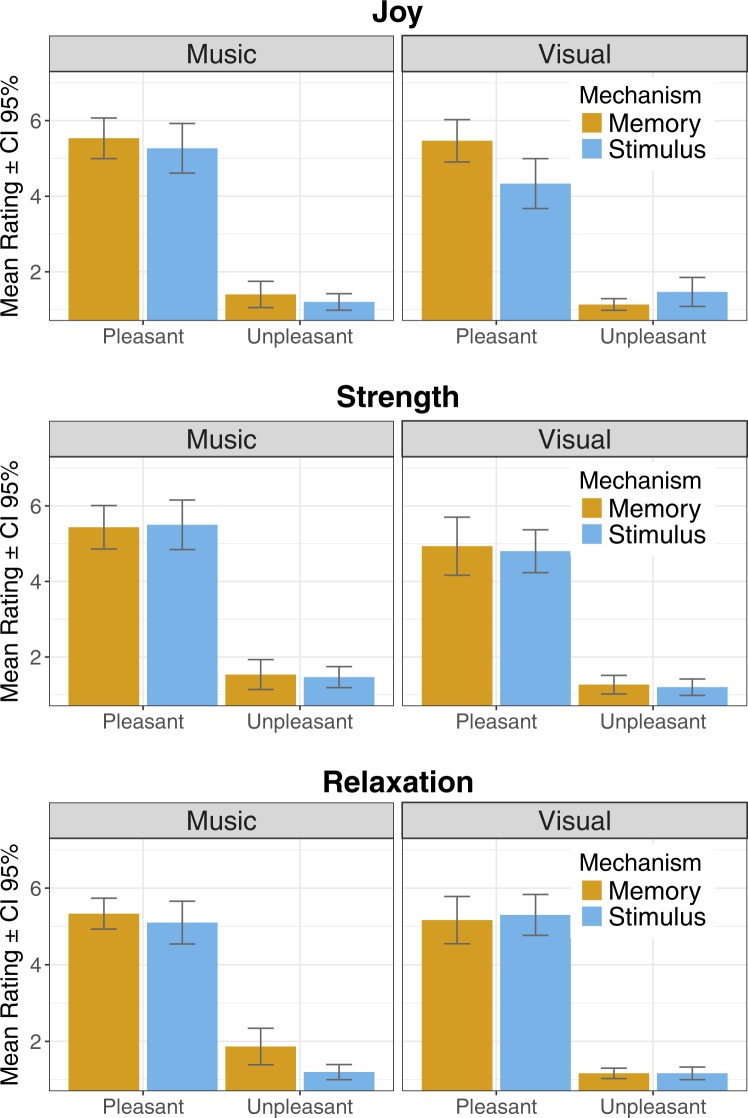
Mean ratings across the Mechanisms for positive emotions (Joy, Strength, and Relaxation).

Over *relaxation*, a significant main effect of *valence* (F (1, 29) = 368.31; p < 0.001) was found, with relaxation being experienced more strongly with pleasant than unpleasant stimuli. A significant interaction of *modality* × *mechanism* (F (1, 29) = 4.67; p = 0.039) was also observed: memory was a more prevalent mechanism in inducing relaxation in musical than in pictorial stimuli. Mean ratings for positive emotions across modality, valence, and mechanism are depicted in Fig. 1.

#### Social emotions

For *kinship*, significant main effects were found for *valence* (F (1, 29) = 135.16; p < 0.001), and *mechanism* (F (1, 29) = 26.77; p < 0.001): kinship was induced particularly by memories and pleasant stimuli. Significant interaction was found for *modality* × *valence* (F (1, 29) = 4.43; p = 0.044) with the prevalence of pleasant stimuli as the source of kinship being slightly more prominent for music than pictures. Significant interaction was also found for *mechanism* × *valence* (F (1, 29) = 11.8; p = 0.002), with the effect of memory inducing kinship being particularly clear when the stimuli were pleasant.

**Figure 2:**
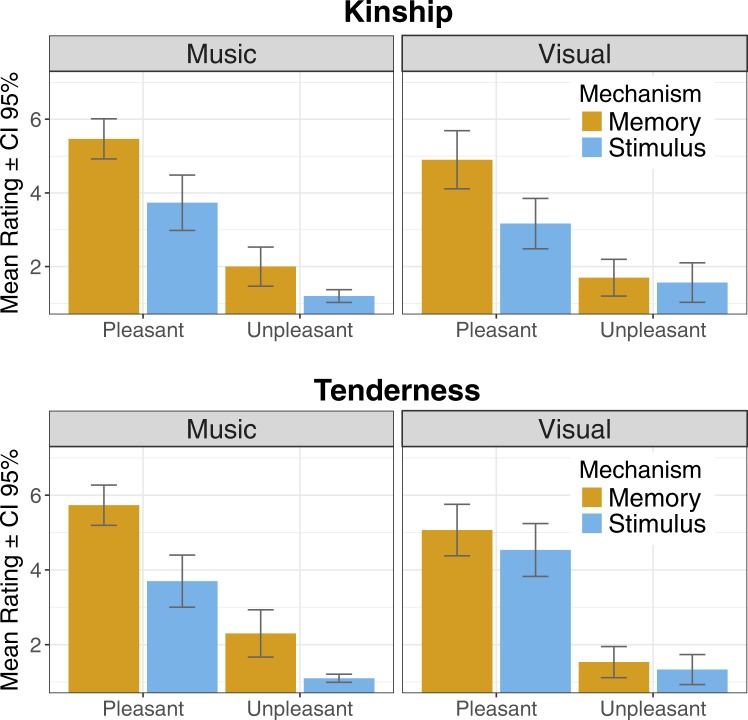
Mean ratings across the Mechanisms for social emotions (Kinship and Tenderness).

Regarding *tenderness*, significant main effects of *valence* (F (1, 29) = 264.86; p < 0.001), and *mechanism* (F (1, 29) = 19.10; p < 0.001) were observed: similarly to kinship, tenderness was particularly induced by memories and by pleasant stimuli. A significant interaction for *modality* × *mechanism* (F (1, 29) = 10.59; p = 0.003) showed that the prevalence of memory as the induction mechanism was more pronounced with musical than pictorial stimuli. Mean ratings for social emotions across modality, valence, and mechanism are depicted in Fig. 2.

#### Negative emotions

Significant main effects for *sadness* were found for all variables: *modality* (F (1, 29) = 6.24; p = 0.018), *valence* (F (1, 29) = 30.39; p < 0.001), and *mechanism* (F (1, 29) = 16,15; p < 0.001). The experience of sadness was generally more prevalent when evoked by pictorial stimuli, and when induced by memories rather than stimulus features. Negative stimuli compounded the occurrence of sadness. Significant interaction was found for *mechanism* × *valence* (F (1, 29) = 20.66; p < 0.001): the impact of memory was particularly prevalent in inducing sadness when the stimuli were unpleasant. A significant 3-way interaction of *modality* × *mechanism* × *valence* (F (1, 29) = 5.03; p = 0.033) indicated that when evoked by pleasant musical stimuli, sadness was induced predominantly through stimulus features, while when evoked by unpleasant musical stimuli or any visual stimuli sadness was prevalently induced by memories

**Figure 3:**
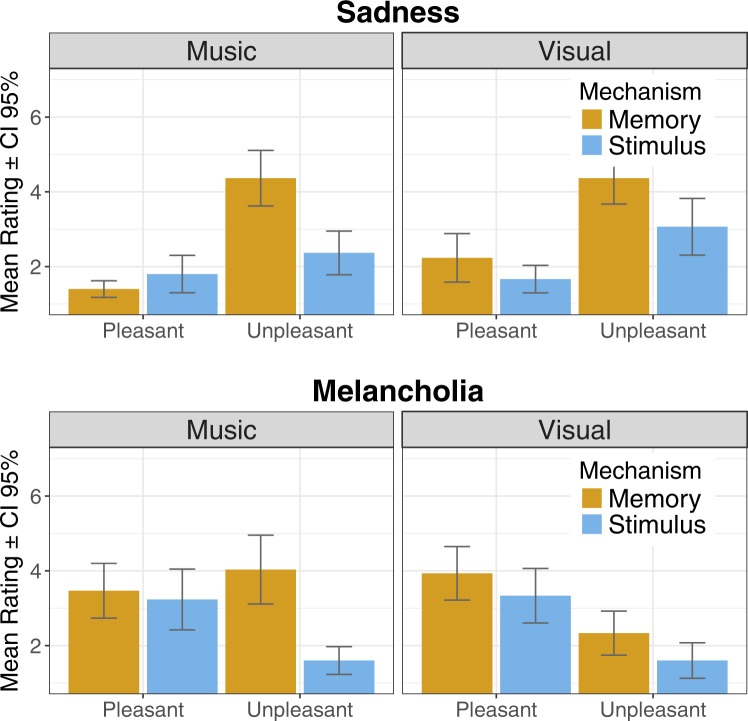
Mean ratings across the Mechanisms for negative emotions (Sadness and Melancholia).

With regards to *melancholia*, significant main effects of *valence* (F (1, 29) = 10.33; p = 0.003), and *mechanism* (F (1, 29) = 24.17; p < 0.001) were found. In contrast to sadness, melancholy was experienced more typically in the context of pleasant stimuli. Similar to sadness, memory was more effective than stimulus features in inducing melancholia. Significant interactions were found for *modality* × *valence* (F (1, 29) = 5.93; p = 0.021), *mechanism* × *valence* (F (1, 29) = 6.07; p = 0.020), and *modality* × *mechanism* × *valence* (F (1, 29) = 4.84; p = 0.036). In pictures, memory was more effective in inducing melancholia than stimulus features regardless of the stimulus’ valence. In contrast, for music, the effect of memory was clear only in the context of unpleasant stimuli. It thus appeared that the role of memory in inducing melancholia was particularly pronounced in unpleasant musical stimuli. Mean ratings for negative emotions across modality, valence, and mechanism are depicted in Fig. 3.

### Results of EEG-measures

The EEG data were utilized to substantiate the effects of emotion induction mechanisms observed in self-evaluations. A repeated-measures ANOVA was executed to examine the possible interactions between Modality, Valence, and Mechanism in the theta oscillations from frontal midline electrodes. In addition, the effects on laterality (left vs. right) on modality, valence and mechanism were explored by looking at the frontal alpha oscillations.

#### Frontomedial theta

**Figure 4:**
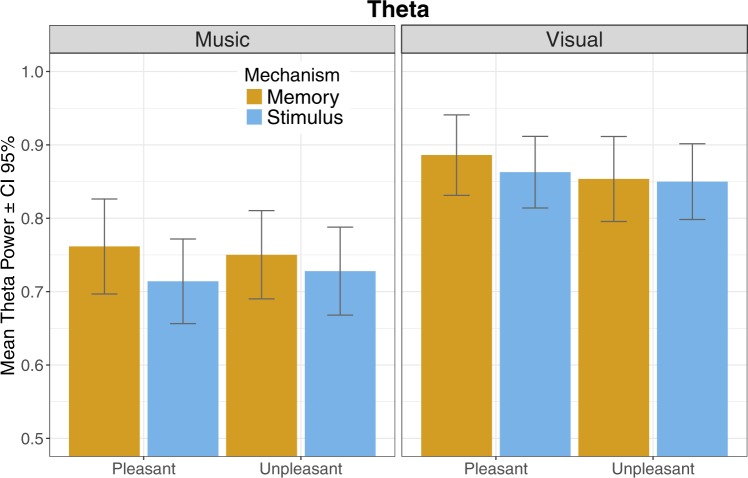
Mean magnitude of theta frequency band of EEG activity.

The main results are largely in support of the behavioral results; The *modality* × *valence* × *mechanism* analysis yielded a significant main effect of mechanism (F (1,28) = 5.10; p < 0.05, ${\eta }_{p}^{2}$ = 0.154), indicating stronger theta activity during memory-related stimulus material, and a significant main effect of modality (F (1,28) = 39.93; p < 0.001; ${\eta }_{p}^{2}$ = 0.588), indicating more theta activity during picture viewing. No significant main effect of valence nor interactions were observed (Fig. 4).

*Valence* × *mechanism* interactions regarding music demonstrated no significant effects, but a trend (p = 0.062, ${\eta }_{p}^{2}$ = 0.119) for more theta activity during memory condition was found. Also, for pictures, *valence* × *mechanism* interactions did not cause a significant effect, but the observed significance level (p = 0.052, ${\eta }_{p}^{2}$ = 0.128) indicate that elevated levels of theta activity might be specifically linked to pleasant memorising.

#### Frontal alpha (central locations omitted in order to evaluate laterality effects)

**Figure 5:**
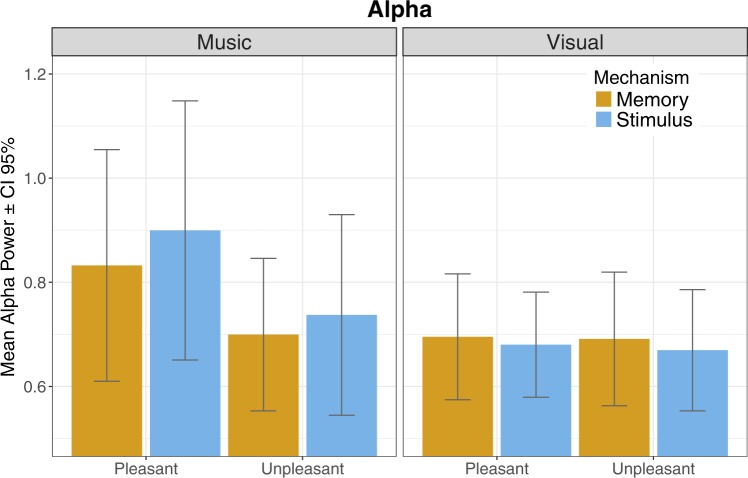
Mean magnitude of alpha frequency band of EEG activity.

For frontal alpha activity, the *hemisphere* × *modality* × *valence* × *mechanism* showed a significant main effect of valence (F (1,28) = 6.09; p < 0.05; p_eta2 = 0.18) indicating stronger alpha oscillations during pleasant events. Also, a significant interaction effect of *modality* × *valence* (F (1,28 = 6.22; P < 0.05; ${\eta }_{p}^{2}$ = 0.18), and *location* × *mechanism* (F (1,28) = 4.61; p < 0.05; ${\eta }_{p}^{2}$ = 0.14) was found, suggesting more alpha oscillations during music (Fig. 5). To further explore the laterality effect of valence, we ran separate analyses comparing F3-F4 and F7-F8 electrode pairs instead of pooled left vs. right signals; results showed no lateral effect on valence.

For music, a significant main effect of valence occurred in *location* × *valence* × *mechanism* interaction (F (1,28) = 6.96; p < 0.05; ${\eta }_{p}^{2}$ = 0.20). There was also an *location* × *valence* interaction approaching significance (F (1,28) = 4.00; *p* = 0.055, ${\eta }_{p}^{2}$ = 0.13). Elevated levels of alpha activity might be specifically linked to lateralisation in specific frontal brain locations during pleasant music. To eliminate the possibility that alpha range oscillations occur due to participants having their eyes closed, the number of eye blinks during pleasant music were calculated (valence: *p* = 0.224; mechanism: *p* = 0.176; *valence* × *mechanism*: *p* = 0.461). The number of eye blinks remained similar throughout distinct conditions. In terms of the number of eye blinks, no differences were found between music and pictures suggesting that pleasant music may, indeed, cause relaxation. The repeated-measures ANOVA on *location* × *valence* × *mechanism* interactions during pictures did not show any significant effects.

## Discussion

This study investigated how affective experiences to pleasant and unpleasant music and pictures vary if induced by personal memories or stimulus features. We particularly tested whether memory, in comparison to stimulus features, would play a major role in inducing different types of emotions (positive, negative, social), and whether this impact of memory would be similar in the case of both music and pictures, and pleasant and unpleasant stimuli. Confirming our hypothesis concerning the prevalence of memory as an induction mechanism, the results showed that the role of memory indeed is indeed central in inducing emotional experiences: stimuli chosen based on memories were more inductive of *joy, kinship, tenderness, sadness*, and *melancholia* than stimuli chosen based on the stimulus features. Only relaxation and strength did not show a main effect of mechanism. Overall, it can be concluded that memory plays an important role in the induction of all types of emotions, but this effect seems to be even more pervasive for the social and negative emotions in comparison to general positive emotions.

Main effects of modality on emotions were not expected, but we did observe that music led to slightly higher ratings of joy and strength, while pictures evoked more sadness than music. Concerning our exploration of the role of memory across the domains, the results generally confirmed that memory is the primary induction mechanism regardless of the stimulus domain. Yet, some distinct asymmetries related to the domain and emotion induction mechanism could also be observed. The pronounced role of memory as an induction mechanism was generally even more evident in music than in pictures. This could reflect the fact that music engagement is closely linked to everyday life events, as music can serve as the background soundtrack for a range of such events. Also, our assumption that memories would play a stronger role for inducing negative affects in music than in pictures received some support: for the induction of melancholia and sadness, memory contributed more highly to these emotions in comparison to stimulus features, and this was particularly clear in the case of unpleasant musical stimuli. Interestingly, the stimulus features were more inductive of sadness in the case of pleasant musical stimuli.

The neural measures confirmed the validity of the setting by indicating the presence of memory processing during the memory conditions: Increased theta oscillations that occurred during the memory condition bolster the earlier observations relating theta to memory tasks^74^. The low arousal pleasant experiences were demonstrated in neural responses as increased alpha oscillations in the memory condition. For instance, pleasant sadness is associated with social signals and functions of music^75^, and the function of distinct neural areas in contrast to those activated by psychological pain, such as posterior cingulate cortex, cerebellum, and parahippocampal gyrus^76^. In future research, however, the study design could capture the effects of observable stimulus features in more detail (e.g. lyrics, genre, familiarity). For further differentiating the types of pleasant and unpleasant experiences based on mechanism and modality, one appealing direction is to alter the conditions to have an effect of the function of particular emotions (e.g. social).

As with all research, the present findings need to be considered in the light of inherent limitations; as participants were exposed to their own choices of music and pictures, it might be argued that it is not the stimulus itself, but the memories associated with it that induced the emotions. More detailed information about the memories could further benefit in the analysis as well. On the one hand, we did not collect data on how much exposure the participants had to the stimuli beforehand, or whether they just thought about this while rating their emotions for the questionnaire. If the latter is true, such retrospective responses can be biased and unreliable. On the other hand, concurrent exposure (Cf.^77^) to the stimuli may create further memories related to the process of filling in the questionnaire, which might influence the experience in various ways (see^74^). To address this, one could for instance utilise the Fading Affect bias to normalize the effects of memories from the distant past^78^. Another option is to ask people to rate the same 4 popular songs and 4 popular pictures and indicate for each whether they have a memory associated with them. This way all participants are being exposed to the same stimuli and any confounds related to familiarity are minimised. One could also carry out correlational research where one predicts the intensity of emotions from scales expressing familiarity and memorability. In the present study, the relatively small sample size and bias in gender distribution restricts the generalization of the results beyond the current sample. While the exclusion of potential confounding variables certainly improves internal validity, further research with larger and more representative samples is certainly warranted before reaching any general conclusions.

Numerous unanswered questions remain in emotion research, particularly concerning mechanisms in general and the complex, social emotions. The present study took a novel step in investigating the interaction between emotion induction mechanisms, emotional valence, and stimulus modality by combining self-reports and objective measures (EEG) using self-selected stimuli. Overall, the present findings suggest that future research on complex emotions should consider the role of memories as a crucial element of the investigation. Deconstructing the central elements of the memories by focussing on familiarity, autobiographical salience, or timeframe of them might be the most effective strategy to pursue this topic (e.g.^51^). Another line of research is to explore in more detail the interactive effects of memories and stimulus properties in paradoxical affects such as the simultaneous experience of pleasantness and sadness. Furthermore, despite the inherent differences in the stimulus properties between images (spatial) and music (temporal), the concurrent use of multiple modalities offers exciting prospects to further our knowledge of affective experiences regardless of the domain.

## Data Availability

The datasets used in this study are published in Harvard Dataverse 10.7910/DVN/ZZR7WX.
